# Cortical Mechanisms of Multisensory Linear Self-motion Perception

**DOI:** 10.1007/s12264-022-00916-8

**Published:** 2022-07-12

**Authors:** Luxin Zhou, Yong Gu

**Affiliations:** 1 CAS Center for Excellence in Brain Science and Intelligence Technology, Institute of Neuroscience, Chinese Academy of Sciences, Shanghai, 200031 China; 2 University of Chinese Academy of Sciences, Beijing, 100049 China

**Keywords:** Multisensory integration, Self-motion perception, Vestibular, Optic flow

## Abstract

Accurate self-motion perception, which is critical for organisms to survive, is a process involving multiple sensory cues. The two most powerful cues are visual (optic flow) and vestibular (inertial motion). Psychophysical studies have indicated that humans and nonhuman primates integrate the two cues to improve the estimation of self-motion direction, often in a statistically Bayesian-optimal way. In the last decade, single-unit recordings in awake, behaving animals have provided valuable neurophysiological data with a high spatial and temporal resolution, giving insight into possible neural mechanisms underlying multisensory self-motion perception. Here, we review these findings, along with new evidence from the most recent studies focusing on the temporal dynamics of signals in different modalities. We show that, in light of new data, conventional thoughts about the cortical mechanisms underlying visuo-vestibular integration for linear self-motion are challenged. We propose that different temporal component signals may mediate different functions, a possibility that requires future studies.

## Introduction

Accurate and precise perception of self-motion is critical for animals’ locomotion and navigation in the three-dimensional (3D) world. For example, in environments where landmark cues are not salient or do not provide reliable information, self-motion cues can be utilized by organisms to maintain navigation efficiency through an algorithm of vector calculation, a process also known as path integration [1, 2].

Self-motion in the environment leads to receiving multiple sensory cues involving vestibular, visual, proprioceptive, somatosensory, and auditory motion. For vision, the movement pattern of the visual background on the retina, known as optic flow [3], is caused by relative motions between an observer and outside scenes, providing a very powerful signal about self-motion status, such as heading direction. Optic flow reflects visual information pooled across the entire field, including the periphery, and thus can be exploited to simulate physical self-motion. In laboratories, the optic flow has been shown to elicit the illusion of self-motion, either rotation or translation of the whole body in the environment; this illusion is called vection [4]. However, relying only on visual cues is not a good strategy. This is because visual cues can be difficult to access in some cases, such as in a dark environment or inclement weather in which visual cues are rare. In addition, visual cues originate from the retina and thus are easily confounded by eye movements [5, 6], head movements [7], and independent object motion that accompanies self-motion [8, 9], all of which generate additional motion vectors on the retina that are irrelevant to the true direction of self-motion. Thus, nonvisual cues could help in these cases. The vestibular system provides such important signals. Specifically, in the inner ear, the otolith organs and semicircular canals are responsible for the detection of linear translation and angular rotation of the head, respectively [10–15]. Immediately after bilateral labyrinthectomy, monkeys could not perform a heading discrimination task when relying only on vestibular input, yet visual performance was largely unaffected. In the next several months, the animals’ heading discriminability in darkness gradually recovered, as reflected by reduced psychophysical thresholds, yet they reached a plateau with a magnitude approximately tenfold larger than that of the control [16]. These data suggest that although the animals could receive some compensatory inputs from other sources like somatosensory input, vestibular cues are the major information source for self-motion direction judgment when visual cues are inaccessible.

The brain makes full use of these available cues for self-motion perception by combining information across sensory modalities. This is useful for a number of purposes, including (1) compensation when one cue is lacking, (2) disambiguation when one cue is compromised, and (3) reduction of noise. In addition, the brain often faces the more difficult task of combining information arising from a common event and segregating irrelevant cues derived from different events. Most of these processes can be described within a theoretical framework of the Bayesian inference model [17], which has received support from numerous psychophysical studies across many sensory domains (for review, see ref. [18]). A Bayesian model has also been successfully applied to visuo-vestibular integration for self-motion in many experiments, supporting the idea that humans, as well as nonhuman primates, can integrate the two cue modalities in a statistically optimal or near-optimal way.

Neural correlates of visuo-vestibular integration have mainly been explored by single-unit recordings in awake, behaving macaques, beginning ~20 years ago [19]. Several cortical areas have been revealed thus far by different laboratories. One obvious criterion for the inclusion of such areas is that their neurons should be modulated by both optic flow and vestibular stimuli. A number of areas meet this criterion, including the extrastriate visual cortex [19, 20] (for reviews, see ref. [21, 22]), posterior parietal area [23], and frontal cortex [24]. These neurophysiological findings provide valuable insight into how neural activity is tuned to self-motion stimuli in both the spatial and temporal domains. In this review, we discuss recent progress in visuo-vestibular integration for self-motion perception. We particularly focus on single-unit activity data collected in cortical areas in animal models under linear self-motion conditions for a number of reasons: (1) Neurophysiological data obtained by recording single-unit activity with high spatial and temporal resolution allow us to address the issues proposed in the current review. Thus, data from imaging studies performed mainly on humans are not used and discussed here. (2) Several cortical areas have been clearly shown to process optic flow signals, whereas it is less clear whether subcortical areas such as the brainstem can process optic flow *per se*. (3) Compared to rotation, a much larger dataset has been acquired under transient linear motion conditions that mainly activate the vestibular otolith system. Specifically, we first summarize behavioral results using an experimental paradigm involving multisensory integration. We then summarize neurophysiological findings in the brain, discussing how they may mediate the behavior. We show that neural evidence from these studies may or may not be consistent. Finally, we propose our hypothesis, showing how recent findings could make sense of a neural circuit that may underlie visuo-vestibular integration for multisensory self-motion perception. Our hypothesis can aid in the design of future studies to further disentangle the functions of cross-modality signals in the brain for locomotion and navigation in the world.

## Behavioral Model System for Multisensory Heading Perception

**Fig. 1:**
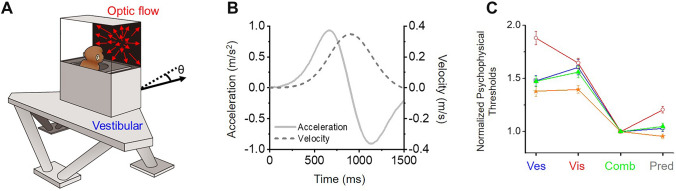
A virtual reality system for studying visuo-vestibular integration. **A** Schematic of the experimental paradigm. The motion platform and visual display provide inertial motion signals and optic flow signals, respectively, to simulate locomotion in the environment. The black arrow indicates the forward motion of the motion base, and *θ* indicates the deviation of motions from straight ahead. **B** Transient motion has a Gaussian-shaped velocity curve, with a biphasic acceleration profile to activate the peripheral vestibular channel. Optic flow follows the same profile. Redrawn using data from ref. [23]. **C** Average normalized psychophysical thresholds across behavioral sessions from four monkeys who discriminated heading directions based on vestibular alone (Ves), visual alone (Vis), and a combination of the two stimuli (Comb). “Pred” indicates the threshold predicted from the Bayesian optimal cue integration theory, which is computed based on thresholds in either single cue conditions (equation 1). In each trial during the heading discrimination task, animals experienced linear forward motion based on either type of stimulus with a small deviation (θ) of the leftward or rightward component. The animals were typically required to maintain central fixation across the stimulus duration (1–2 s). At the end of each trial, the central fixation point disappeared, providing a “go” signal. The animals made saccadic eye movements to one of the two choice targets presented on each side of the visual display to report their experienced heading direction. A correct choice would lead to a reward. Redrawn using data with permission from ref. [23, 32].

One important component of self-motion perception is heading perception, in which the individual detects the instantaneous direction of the head or whole body in space during spatial navigation. Accurate and precise heading estimates are critical to correctly guide forward motion in animals (e.g., to locate prey or escape from predators) and humans (e.g., to drive vehicles and play sports such as skiing). In the laboratory, using a motion platform or vehicle to provide real vestibular input and a visual display to provide optic flow (Fig. 1A, B), numerous studies have shown that humans can integrate optic flow and vestibular cues to improve heading judgments compared to what they can derive under either single-cue condition [25–31]. Similar results have been found in nonhuman primates when the animals were trained to discriminate heading directions by making saccades to alternative choice targets to indicate perceived heading (e.g., left *vs* right relative to straight forward, Fig. 1C) [32–34]. Interestingly, in both humans and monkeys, the amount of behavioral improvement is close to that predicted by Bayesian optimal integration theory when both visual and vestibular cues are provided to the subjects [18, 35]. According to this theory, a maximum multisensory integration benefit would be achieved when the two cues share the same reliability. This is indeed what most studies have attempted to achieve. In particular, the reliability levels of the two heading cues are adjusted to generate roughly equal performance quality (e.g., discrimination threshold, *σ*; see equation 1). In this case, the largest cue combination effect is typically seen with the discrimination threshold reduced by a factor of 1/$\surd 2$. That is, *σ*_combined_ = *σ*_visual or vestibular_/$\surd 2$.

1 $$ \sigma_{{{\text{combined}}}} = \sqrt {\frac{{\sigma_{{{\text{vestibular}}}}^{2} \times \sigma_{{{\text{visual}}}}^{2} }}{{\sigma_{{{\text{vestibular}}}}^{2} + \sigma_{{{\text{visual}}}}^{2} }}} $$

Using a motion platform virtual reality system, it is convenient to manipulate the congruency of the visuo-vestibular inputs. In contrast to the above experiments in which spatially congruent cues are provided to examine the psychophysical threshold, a conflict of heading directions indicated by different cues is also purposely introduced together with varying reliability of the two cues. Under the condition of conflicting cues, such studies examine how heading perception is biased, for example, toward the more reliable cue, as reflected by a shift in the mean estimate (*µ*) or the point of subjective equality (PSE) in the psychometric function (see equation 2). This has been found to be the case in such studies [25, 28, 36]. Note, however, that in some cases, the measured weight, as reflected by the shifted *µ* or PSE, could be away from the prediction based on the model [25, 36, 37]. This may require the inclusion of a prior term. Indeed, a model including such a prior favoring vestibular input does explain the data much better than a model without such a term [25, 36].

2 $$ \mu_{{{\text{combined}}}} { } = { }\frac{{\sigma_{{{\text{vestibular}}}}^{2} }}{{\sigma_{{{\text{visual}}}}^{2} + \sigma_{vestibular}^{2} }}\mu_{{{\text{visual}}}} + \frac{{\sigma_{{{\text{visual}}}}^{2} }}{{\sigma_{{{\text{visual}}}}^{2} + \sigma_{{{\text{vestibular}}}}^{2} }}\mu_{{{\text{vestibular}}}} $$

Together, these psychophysical studies demonstrate that human and nonhuman primates can integrate optic flow and vestibular cues to improve heading estimates in a way that is consistent with predictions from Bayesian optimal integration theory. Among these successful model systems, the nonhuman primate system stands out, as illustrated in the following sections; it is an ideal model for studying single-unit-resolution physiology that will provide great insight into neural mechanisms underlying the behavior.

## Neurophysiology: Spatial Modulations

### Passive-Fixation-Only TASK

The first step to explore neural correlates underlying multisensory heading perception is to measure basic tuning functions in response to optic flow and vestibular stimuli. Pioneering studies were conducted by Duffy and colleagues [19, 38]. Using a two-dimensional (2D) motion platform that can physically translate subjects (e.g., monkeys) in the horizontal plane, the researchers systematically investigated single-unit activity in the dorsal portion of the medial superior temporal sulcus (MSTd) in the dorsal visual pathway. The monkeys merely need to maintain fixation while passively experiencing stimuli. The researchers found that in addition to optic flow [39–41], approximately one-third of MSTd neurons are also modulated by vestibular stimuli even in total darkness. Later, Angelaki, DeAngelis, and colleagues developed a virtual reality system with a six-degree-of-freedom motion platform that can either translate or rotate subjects in 3D space, providing a more thorough measurement of neuronal tuning functions in sensory cortices [20]. Using this system, the researchers discovered that nearly two-thirds of MSTd neurons are modulated by both optic flow and vestibular stimuli. The larger proportion of bimodal neurons discovered in this study may be due to the larger acceleration stimulus used (~0.1 G) compared to that used in a previous study (~0.03 G). Whether using a larger acceleration profile would activate even more vestibular-modulated neurons is currently unknown due to the mechanical limitations of these motion platforms. To confirm that the responses recorded under vestibular conditions are truly “vestibular” in origin, the researchers conducted a number of control experiments, such as a total darkness condition to rule out the possibility of retinal slip due to residual light. Importantly, damaging the peripheral vestibular organs diminishes the vestibular responses in the MSTd, providing solid evidence supporting a vestibular-origin hypothesis [16, 42].

A number of key properties have thus been revealed. First, in the traditional visual area of the MSTd, a large proportion of neurons (~2/3) also exhibit significant vestibular responses, while the majority (~95%) of the neurons are modulated by optic flow. Thus, the MSTd is a multisensory area *per se*. Second, preferred linear translation directions for both modalities are widely distributed in 3D space, yet there is a bias toward leftward and rightward motion in the horizontal plane. Computational modeling suggests that this property is ideal for discrimination of heading directions that deviate from straight ahead [43], making the MSTd a suitable neural basis for heading estimation. Third, the preferred heading directions for bimodal MSTd neurons tend to be either congruent or opposite. Neurons with intermediate differences in preferred direction between the two modalities are few, suggesting that the two main subpopulations of neurons execute distinct functions. Intuitively, congruent neurons are activated by consistent visuo-vestibular stimuli and should be activated especially during natural locomotion or navigation. For example, a leftward heading results in rightward motion of optic flow projected on the retina that indicates leftward physical self-motion, exactly matching the vestibular signals. This is indeed the case: congruent neurons tend to show stronger tuning to bimodal stimuli than to stimuli of either single modality. In contrast, opposite neurons show reduced activity under bimodal stimuli, which should not be able to account for cue integration. This rationale is confirmed by computational modeling with a selective readout mechanism in which higher weight is assigned to congruent neurons than to opposite neurons [44]. However, what is the purpose of opposite neurons, especially considering their large population? A number of recent studies propose that congruent and opposite neurons could be used for integration and segregation of cues, respectively, according to whether they arise from a common source or different sources [45, 46]. For example, independently-moving objects are frequently encountered during locomotion or navigation. If they are moving in directions that are inconsistent with self-motion, opposite cells are presumably activated. Thus, joint coding by opposite neurons and congruent neurons could be used to judge the true direction of self-motion by parsing out external object motion [47, 48] or judge object motion by removing the self-motion signals [49].

In addition to the MSTd, similar experimental paradigms have been used to screen other cortical areas. Some of the areas show similar properties in that they show both vestibular and optic flow signals, thus serving as potential candidates for multisensory heading perception. These areas include the ventral intraparietal area (VIP) [34, 50–55], the visual posterior Sylvian area (VPS) [56], the smooth pursuit area of the frontal eye field (FEFsem) [57], the posterior superior temporal polysensory area [58], area 7a [59], and the cerebellar nodulus and uvula [60]. Among these, the MSTd, VIP, and FEFsem show fairly strong heading modulations in response to both modalities, and, interestingly, the pattern of coexistence of congruent and opposite cells is similar across areas. The VPS also shows robust vestibular and optic flow signals, yet, surprisingly, most of its bimodal cells are opposite neurons (for review, see ref. [22]).

A number of cortical areas show responses to only one modality; thus, they are not considered multisensory areas. The middle temporal area (MT) and visual area V6 are tuned exclusively to optic flow and are not significantly modulated by vestibular input [61, 62]. Thus, MT and V6 can be classified as traditional visual areas. In contrast, the parieto-insular vestibular cortex (PIVC) is tuned exclusively to vestibular stimuli; global optic flow has no clear modulatory effect on it [63]. Similarly, the posterior cingulate cortex (PCC) also contains robust vestibular signals, while its optic flow signals are much weaker [64], although studies based on imaging do reveal significant optic flow signals in this region (for review, see ref. [65]). Therefore, the PIVC and PCC can be classified as vestibular-dominant areas.

### Heading Discrimination Task

While basic tuning functions measured in passive-fixation-only experiments identify potential candidates for visuo-vestibular integration, experiments with discrimination tasks provide more insight into their roles in multisensory heading perception. Using the same motion platform system, Gu *et al*. trained monkeys to perform a heading discrimination task in which heading directions are varied in fine steps around the reference of straight forward [16]. The animals must correctly judge whether their experienced heading direction, based on vestibular input, optic flow, or a combination of the two, is leftward or rightward by making saccadic reports to one of the two choice targets that are presented on either side of the visual display. In the two-alternative forced-choice (2-AFC) paradigm, only correct answers lead to reward [32]. After training, the animals could typically discriminate heading that deviated from straight ahead by as little as a few degrees based on either single cue. Importantly, when presented with bimodal stimuli, the animals could integrate the two cues to improve discriminability, and the quantity of improvement was close to the amount predicted by the optimal integration theory [32, 34, 36].

Neuronal activity is measured under identical stimulus conditions simultaneously when the animals perform the discrimination task. Thus, by receiver operating characteristic (ROC) analysis, neural signals can be decoded in such a way that they can be directly compared with the behavior [66]. Briefly, an ideal observer discriminates each pair of heading directions based on firing distributions from a single neuron using the ROC algorithm. A full psychometric function is constructed based on all pairs of heading stimuli to quantify the decoder’s discriminability, which can be compared to the animal’s performance. Importantly, covariation of neuronal activity and the animals’ perceptual judgment on a trial-by-trial basis can be measured to imply possible functional links of the neurons in the task [67]. Recordings in the MSTd do reveal that many neurons are modulated by headings varying within a small range (~±10°) from straight ahead, and this modulation can be further strengthened by congruent optic flow and vestibular stimuli [32]. ROC analysis suggests that some individual neurons rival the sensitivity of behavioral performance, yet many others do not. Population analysis assigning a higher weight to congruent visuo-vestibular cells can reproduce the combination effects seen in behavioral data [44]. Interestingly, congruent cells, but not opposite cells, exhibit significant trial-to-trial covariation with the animal’s heading judgment [16, 32]. Note, however, that only spatially-congruent visual and vestibular cues are provided in these studies. Thus, under conditions when vision motion is inconsistent with self-motion [46, 68], opposite neurons are presumably activated, and whether they would exhibit trial-to-trial covariation with the behavior remains an interesting question in need of further study. In any case, all these results appear to support the hypothesis that polysensory areas such as the MSTd are the neural basis of visuo-vestibular integration for heading perception. Similar results have been revealed in other areas such as the VIP [34]. However, as we discuss below, newly-emerging evidence has begun to challenge this idea.

## New Insight from the Perspective of Temporal Dynamics

### Temporal Dynamics in Sensory Cortices

**Fig. 2:**
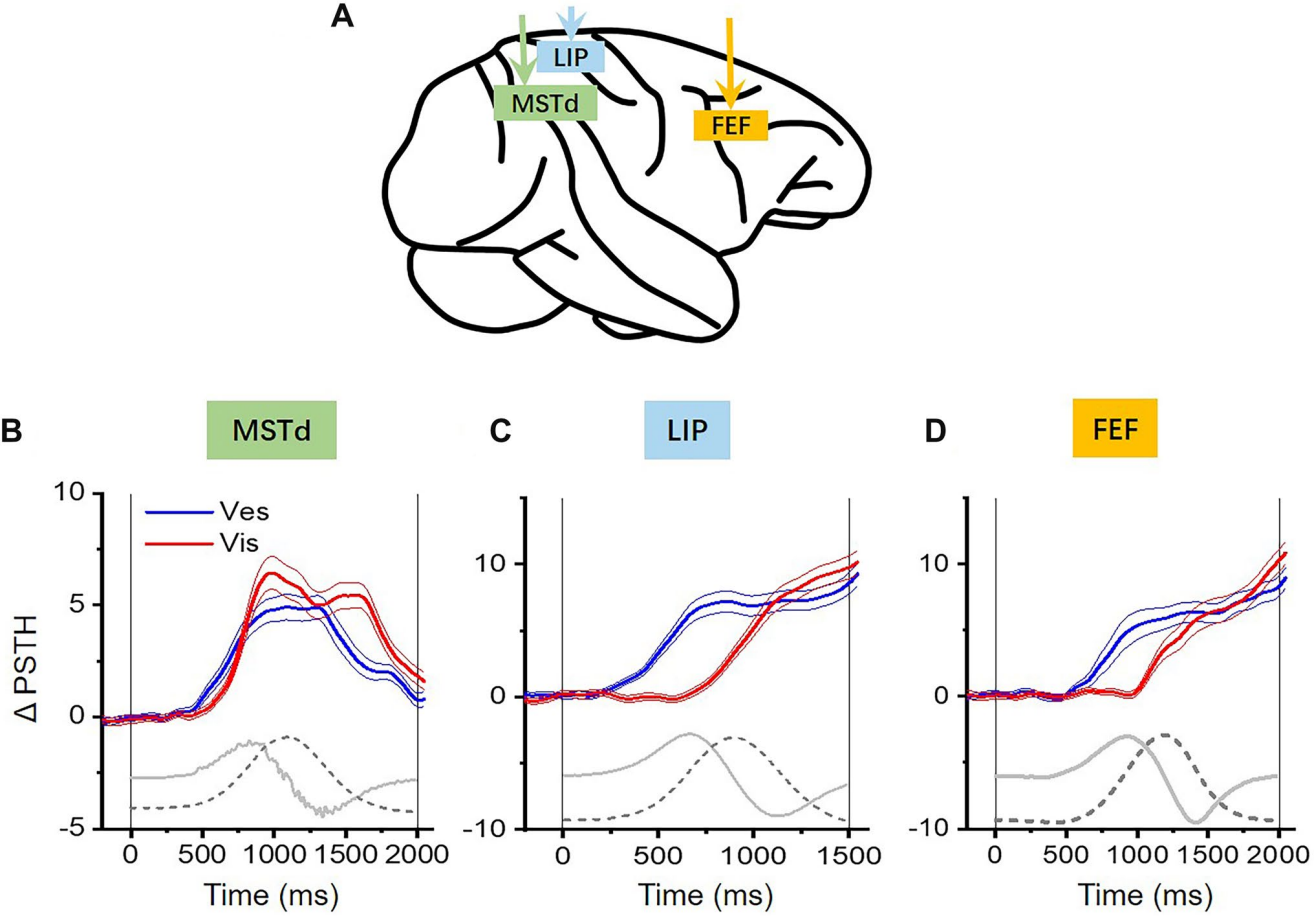
Temporal dynamics of vestibular and visual responses across cortical areas. **A** Schematic of the MSTd in the extrastriate visual cortex, LIP in the parietal cortex, and FEF in the frontal cortex. **B–D** Time course of population average responses to leftward and rightward heading directions quantified by Δ firing rates (ΔPSTH) in the three areas. Experimental conditions were almost identical in these studies, yet the location of choice targets was a bit different across studies. In the MSTd, choice targets were always aligned in the horizontal meridian [32]. In the LIP [23] and FEF [24], choice targets were placed at locations matching the response field of the recorded neurons since these areas are more influenced by the preparation and execution of the saccadic response. Thus, different from the MSTd, the time-course of neuronal activity in the LIP and FEF typically reflects mixed signals of sensory, sensory accumulation, sensory-motor transformation (choice), and motor execution. Solid and dashed gray curves represent the acceleration and velocity profiles of the motion stimulus, respectively. Redrawn using data with permission from ref. [23, 24, 32]. MSTd, medial superior temporal sulcus; LIP, lateral intraparietal area; FEF, frontal eye field.

In the brain, visual motion signals are mainly coded in terms of velocity by individual neurons [20, 69–72], which is the main reason that a constant velocity profile is often used in visual motion studies. In the vestibular system, however, the brain encodes much more complicated temporal dynamics. Although the peripheral otolith organs predominantly encode acceleration of the head, the acceleration signals are integrated to different extents when propagating from the peripheral to the central nervous system, generating a range of temporal dynamics from acceleration-dominant to velocity-dominant profiles, including intermediate patterns [22, 73]. For example, the temporal dynamics of neurons in a number of cortical areas are categorized as “single-peaked” or “double-peaked” according to the number of distinct peaks in the peristimulus time histogram [20, 54–57, 63]. In these studies, a transient, varied velocity profile (e.g., Gaussian), corresponding to biphasic acceleration, is typically provided for the physical motion condition to activate the vestibular peripheral organs. The same motion profile is also used under the visual motion condition to simulate the patterns on the retina during self-motion in the environment. Thus, “single-peaked” and “double-peaked” cells are thought to be velocity-dominant and acceleration-dominant cells, respectively. Across areas, there are trends toward a gradual increase in the proportion of velocity-dominant cells and a gradual decrease in the proportion of acceleration-dominant cells from the PIVC to the VPS, FEFsem, VIP, and MSTd, which implies possible information flow [54]. Furthermore, Laurens and colleagues developed a 3D spatiotemporal model to fit data across spatial directions and time simultaneously, revealing representations of many temporal variables, including velocity, acceleration, and jerk (the derivative of acceleration), in sensory cortices [73]. Recently, Liu and colleagues used a similar method and also discovered that many temporal components of vestibular signals are represented in the PCC [64]. All these studies demonstrate that vestibular temporal dynamics are widely distributed in the brain. Interestingly, it is notable that although the majority of sensory cortices contain a balanced mix of acceleration and velocity vestibular signals, MSTd neurons mainly have a velocity-like component, which is also true of its counterpart in the visual pathway [20, 32]. Thus, vestibular and visual motion responses reach a peak at approximately the same time in the MSTd (Fig. 2A, B), which could facilitate cue integration. This fact appears to support the concept that the MSTd may be an ideal neural basis for visuo-vestibular integration for heading estimates.

### Temporal Dynamics in Decision-related Areas

However, recent studies in higher-level areas, such as the posterior parietal cortex, reveal a different picture. Using the same heading discrimination task with an identical experimental setup to the one used in the above MSTd study, a recent study investigated vestibular and visual signals in the lateral intraparietal area (LIP) [23]. In macaques, the LIP is associated with oculomotor decision-making. Unlike sensory areas (such as the MT) in which neural activity typically follows the stimulus profile, the LIP is thought to accumulate momentary evidence, reflected by a ramping of activity over time. In 2-AFC tasks, ramping can be either up or down, depending on whether the animal’s upcoming choice is toward the response field of the recorded neuron or in the opposite direction [67, 74, 75] (for review, see ref. [76]). Consistent with previous findings, Hou and colleagues found that optic flow-evoked responses in the LIP of monkeys gradually increase or decrease in a manner that predicts the monkey’s upcoming choice [23]. The choice-dependent divergence of neural activity is proportional to the value of velocity. That is, the choice-dependent divergence signal ramps fastest around the peak time of the Gaussian velocity profile, which is also consistent with the idea that the visual system mainly encodes velocity (Fig. 2C).

Similar to optic flow, LIP activity also ramps in the vestibular condition, yet, surprisingly, it is aligned with acceleration instead of velocity (Fig. 2C). This finding is unexpected if the LIP accumulates vestibular evidence from the upstream MSTd. To verify that LIP gathers information on vestibular acceleration and visual speed *per se*, the motion profile was modified and compared. In particular, the bandwidth of the Gaussian velocity profile was varied such that the velocity peak time was unchanged, while the acceleration peak time was shifted. The vestibular ramping activity in the LIP was found to shift accordingly, while visual ramping activity remained the same in the temporal domain. Therefore, inconsistent with input from the MSTd, the LIP selectively collects vestibular acceleration and visual speed information for heading estimation (Fig. 2B, C). Since the acceleration component occurs earlier than the speed, this implies that an animal may make faster decisions about heading directions based on vestibular acceleration under the vestibular condition than based on visual speed under the visual condition. Previous studies, however, used a fixed-duration task that does not allow verification of this hypothesis. In a recent human psychophysical experiment examining reaction time, researchers discovered that subjects tend to make decisions about heading with a reaction time close to the acceleration peak time under the vestibular condition and close to the speed peak time under the visual condition [77]. This behavioral result thus appears to be consistent with findings in the macaque LIP rather than the MSTd.

How does the brain collect and integrate visuo-vestibular signals for heading estimation? Based on the neurophysiological findings in the MSTd and LIP, two alternative models can be proposed. Similar to the two spatial models (congruent and opposite cells) described in the previous sections, we define two alternative models in the temporal domain. In the temporal–congruent model that is based on findings in the MSTd, the brain integrates vestibular and visual speed information to estimate heading. In contrast, in the temporal–incongruent model based on findings in the LIP, the brain integrates vestibular acceleration and visual speed information. In the latter model, because acceleration mathematically peaks earlier than velocity, this implies that vestibular activity would rise earlier than visual activity. This is indeed the case in the LIP, where visual responses lag behind vestibular responses by nearly 400 ms [23].

### Manipulation of Temporal Offset Between Visuo-vestibular Inputs

**Fig. 3:**
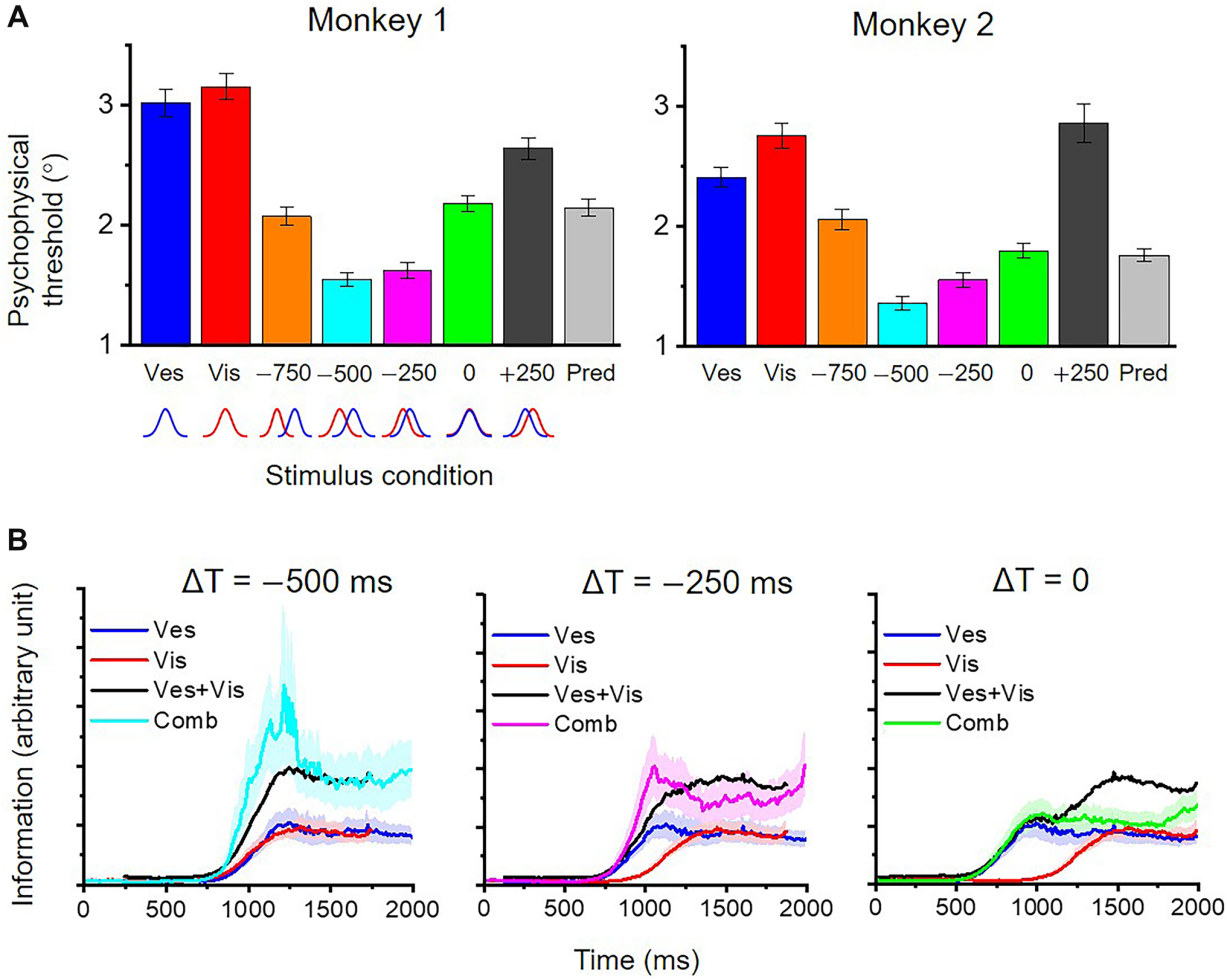
Manipulating temporal offset for visuo-vestibular input. **A** Average psychophysical thresholds for two animals in the two unimodal and five bimodal stimuli conditions with different temporal offsets (ΔT; −750, −500, −250, 0, and 250 ms) between vestibular and visual inputs in the heading discrimination task that is described in the previous section (e.g., Fig. 1C). **B** From left to right: time course of population Fisher information in the FEF under different temporal offset conditions. Ves+Vis represents predictions from a straight sum algorithm based on the two single cue inputs. Comb represents the measured information under bimodal stimuli conditions. Redrawn using data with permission from ref. [24].

To test which model (temporal–congruent *vs* temporal–incongruent) is applied by the brain to perform the heading discrimination task, a recent study by Zheng and colleagues manipulated the temporal synchrony of the visual and vestibular inputs while measuring the animals’ behavioral performance using behavioral paradigms similar to those described in the above section [6]. Specifically, visual input was adjusted to lead vestibular cues with a number of different offsets at steps of 250 ms. The researchers found that, compared with zero-offset conditions that have been conducted in previous experiments [25, 28, 32, 34, 36], the animals performed heading discrimination tasks better when visual cues led vestibular cues by 250–500 ms. Other offsets did not work. For example, if the visual signals led by 750 ms or lagged by 250 ms, performance was reduced. Thus, the offset must be in the specific range of 250–500 ms to increase the benefit of cue integration (Fig. 3A).

Why does a visual-leading offset of 250–500 ms help enlarge the visuo-vestibular integration effect for heading estimation? To explore its neural correlates, the researchers recorded single-unit activity in the LIP, as well as the saccadic area of the FEF (FEFsac), while the animals performed the heading discrimination task with different temporal offset conditions [24]. It has been shown previously that FEFsac in the frontal cortex is analogous to the LIP in the parietal cortex in many ways with respect to their roles in oculomotor decision-making tasks [78–81]. Zheng and colleagues found that FEFsac exhibited properties very similar to the LIP with respect to the accumulation of evidence from optic flow and vestibular input [24]. That is, visual and vestibular ramping activities in the two areas are proportional to velocity (speed) and acceleration, respectively, leading to delayed visual responses that lag vestibular by several hundreds of milliseconds under zero-offset conditions (Figs 2D, 3B). Under temporally manipulated conditions where visual input was artificially adjusted to occur 250–500 ms earlier, visual and vestibular signals in the FEFsac and LIP were aligned more synchronously than under zero-offset conditions (Fig. 3B). To address how these neuronal properties may mediate behavior, the researchers computed population Fisher information to assess the upper bound of heading capacity based on all the recorded neurons [43]. Heading information under the nonzero-offset conditions (Fig. 3B, left and middle panels) was significantly higher around the vestibular peak time than under the zero-offset condition (Fig. 3B, right panel). In some cases, the enhanced heading information (Comb in Fig. 3B) could be even higher than a straight sum prediction (Ves+Vis in Fig. 3B) based on the two single cues, suggesting that some nonlinear gain effect might exist in the FEF or LIP. Thus, reading out information around the vestibular peak time could generate a behavioral pattern across different bimodal conditions, as seen experimentally. In contrast, the MSTd model with temporally-matched visual and vestibular response profiles under zero-offset conditions would predict reduced cue combination enhancement under nonzero-offset conditions, which is unlikely to account for the observed behavior.

## Causality Consideration

In the above sections, all the physiological results are based on a correlation metric. For example, although robust optic flow and vestibular signals are present in many areas such as MSTd, it is unclear whether these signals are truly decoded by downstream areas for task performance. Even if trial-to-trial choice correlations are observed, it is debated whether these signals are truly driven by sensory input (bottom-up) or only by feedback from higher-level areas (top-down). Thus, in addition to these correlation metric measurements, experiments involving causal manipulation provide additional and useful insight in terms of clarifying the neural basis for behavior. Two alternative, complementary methods are typically used by researchers to manipulate neuronal activity in a certain region: inactivation and activation. In primate neurophysiology, inactivation often involves lesions or reversible inactivation by drugs such as muscimol, while activation often involves electrical microstimulation. Specifically, lesion or muscimol inactivation experiments eliminate or suppress neural activity and examine the necessity of the target region for a particular process, which can be reflected by changes in the psychophysical threshold or the overall correct response rate of the animal’s task performance [82]. In contrast, electrical microstimulation activates a clustered population of neurons, introducing an artificial signal into the decision circuit to bias the animal’s perception, which can be reflected by a shift in the PSE in the psychometric functions [83]. Thus, microstimulation examines the sufficiency of target neurons or areas for perception. Note, however, that the two techniques have both advantages and limitations; therefore, it is better to combine the results from the two methods to obtain a more complete picture.

Gu and colleagues used muscimol inactivation and electrical microstimulation to artificially perturb MSTd activity while monkeys performed a visuo-vestibular heading discrimination task [84]. Researchers found that muscimol injection into bilateral MSTd dramatically reduced the animals’ discrimination ability by several times based on optic flow, demonstrating that visual signals in this area are critical for heading perception. However, inactivation effects, while statistically significant, were fairly weak under vestibular conditions, as reflected by an increase in the psychophysical threshold by an average of 10%. Microstimulation in the MSTd produced similar results. While microstimulation significantly biased the PSE under visual conditions (also see ref. [85, 86]), there was no effect in the vestibular condition.

Similar experiments have been conducted by extending the same method to other areas. In particular, Chen and colleagues applied muscimol to inactivate a number of areas, including the PIVC, VIP, and MSTd [87]. While researchers found the same result in the MSTd as in the previous study, they found something interesting in the other two areas. First, injecting muscimol bilaterally into the PIVC strongly worsened the animals’ heading performance under vestibular conditions, a result that is in sharp contrast to the effect in the MSTd [87]. Thus, unlike the vestibular signals in the extrastriate visual cortex (MSTd), vestibular signals in the vestibular cortex (PIVC) are critical for heading estimation based on vestibular cues. Second, inactivation of the VIP did not generate any significant effects in either visual or vestibular conditions. This is surprising because the VIP contains robust visual and vestibular signals. More surprisingly, VIP activity has been shown to be tightly correlated with the animal’s perceptual choice on a trial-by-trial basis [34, 86]. Therefore, visual and vestibular signals in the VIP may not be critical, at least in the context described in the above studies.

In summary, the results from causal manipulation experiments suggest that in the context of a fine heading discrimination task, vestibular signals in the PIVC, but not in the dorsal visual pathway (such as the MSTd and VIP), are critical for perceptual judgment. In contrast, motion signals in the dorsal visual pathway, including the MT [85, 86] and MSTd, are critical for heading judgment.

### Perspectives

**Fig. 4:**
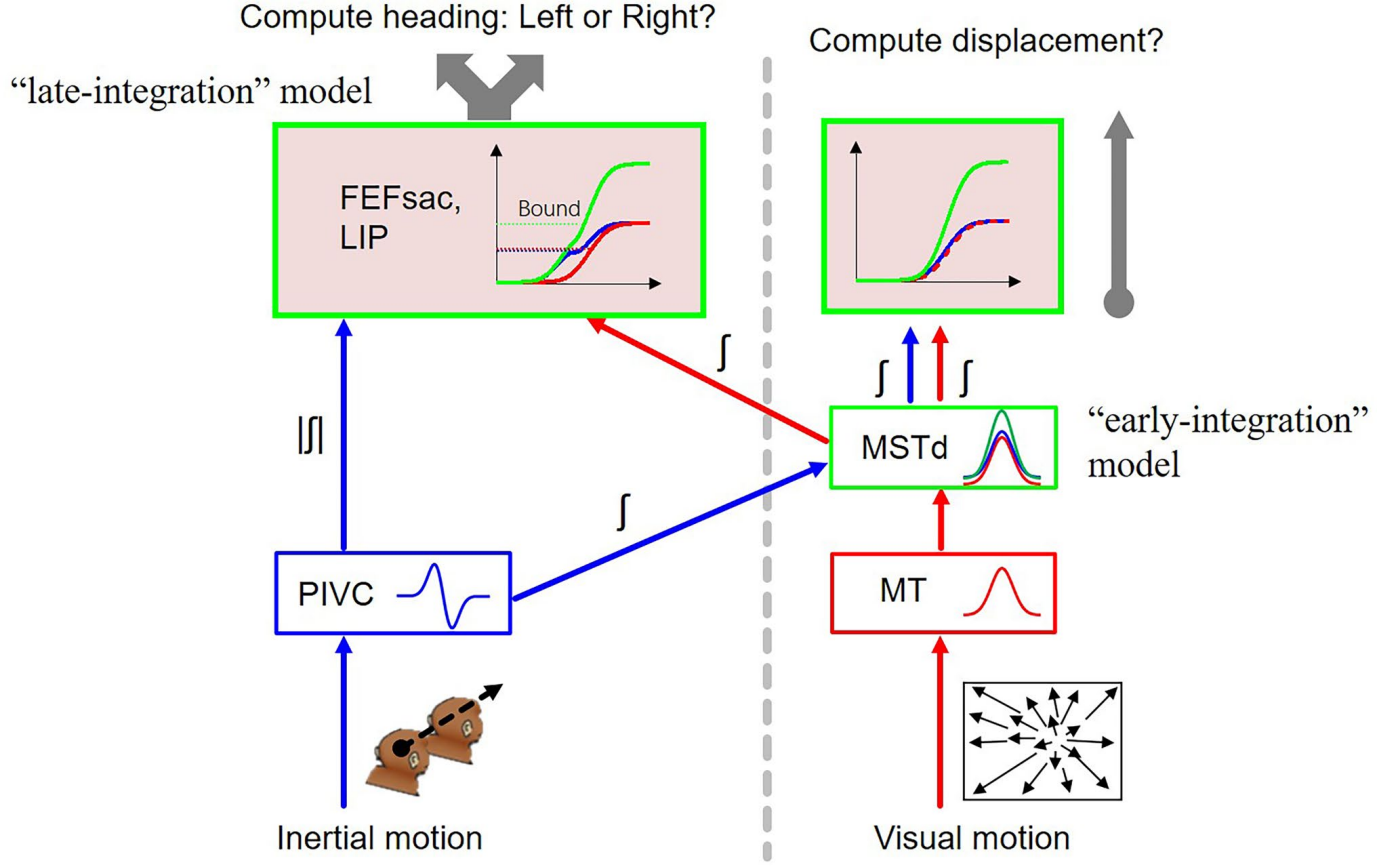
Hypothetical neural circuits mediating visuo-vestibular heading perception. To estimate heading, vestibular acceleration and visual speed signals are hypothesized to propagate through individual pathways, for example, the PIVC and extrastriate visual cortex, respectively, to high-level decision-related areas including sites in the frontal lobe and posterior parietal lobe (late convergence/integration pathway). In these areas, each type of momentary sensory evidence is accumulated and can be described by a drift-diffusion model (see ref. [90]) (blue and red curves). Cross-modality sensory evidence is also integrated in these areas (green curve). Alternatively, the previously observed convergence of two signals in the sensory area MSTd (early convergence/integration pathway) may be involved in other contexts, for example, computing displacement (panel on the right side of the vertical dashed line). Briefly, vestibular and visual signals with speed temporal profiles converge and are integrated in the MSTd. These signals are then transmitted to decision-related areas for evidence accumulation and decision formation. FEFsac, saccadic area of the frontal eye field; LIP, lateral intraparietal area; MSTd, medial superior temporal sulcus; MT, middle temporal area; PIVC, parieto-insular vestibular cortex.

Regarding the stage at which multisensory information converges, a few possibilities exist [88]. In the late-integration model, signals of different modalities are propagated to the parietal and frontal cortices along each sensory channel. Sensory evidence is accumulated and integrated across modalities for decision-making. Candidates for these areas include the LIP and FEFsac in which researchers have discovered mixed signals of sensory, sensory accumulation, sensory-motor transformation (choice), and motor execution. In the early-integration model, different modality signals are first gathered and integrated at earlier stages (such as the MSTd) before they are further propagated to parietal and frontal cortices for decision-making. Which model is true for multisensory heading perception? As we have discussed in this review, we propose that despite earlier studies suggesting that the MSTd contains robust vestibular and optic flow signals, the two signals may not be integrated here for heading perception, based on new evidence from temporal- and causal-manipulation experiments. Instead, these new pieces of evidence support the idea that multisensory heading perception is likely to follow the late-integration model. In particular, vestibular information may be propagated through the PIVC, which is a vestibular-dominant area with many acceleration components [63]. Optic flow signals are fairly weak in this area [63, 87], although responses evoked by large-field optokinetic stimuli have been recorded for the majority of neurons [89]. In contrast, optic flow signals are mainly transmitted through the classical dorsal visual pathway, including the MT and MSTd. The two heading signals in each sensory pathway finally converge at high-level decision-related areas, including the frontal and posterior parietal decision lobes, for evidence accumulation and integration (Fig. 4, left panel). In conclusion, we propose that multisensory heading perception may occur through a late-integration mechanism rather than an early-integration mechanism.

If visuo-vestibular heading perception is based on the late-integration model by which only optic flow and not vestibular information in the MSTd is applied for direction judgment, an obvious question arises: for what purpose are the vestibular signals in the MSTd used? With respect to velocity-dominant vestibular signals in this area [20, 32, 73], we speculate here that these signals may be temporally integrated to estimate the displacement of the head in the environment. For example, during locomotion, the brain needs to update displacement to maintain high-quality tracking of visual targets that move in the field. In navigation, the velocity signal could also be used to estimate the distance traveled. In these cases, vestibular signals, originating from the periphery and encoding acceleration, are temporally integrated twice to estimate displacement or distance [91, 92]. It has been shown that optic flow signals are also useful for distance estimation [91–93]. However, distance estimates based on either cue are limited, especially in the sense that they accumulate errors over time [94]. Thus, the coexistence of visuo-vestibular signals with temporally-matched kinetics in the MSTd may be ideal to fulfill these functions. In this sense, unlike heading perception, which is proposed to be mediated by the late-integration model, head or whole-body displacement perception may be mediated by the early-integration model.

In summary, the neural mechanisms underlying visuo-vestibular integration for self-motion perception have been investigated for many years. Much attention has been paid to spatial-modulation properties, which provide valuable insight into our understanding of how the brain integrates multisensory information to strengthen the perception. Recent studies, however, from the perspective of temporal properties, have yielded new findings that drive us to revise our conventional thoughts. The fact that vestibular temporal dynamics include different components in the brain suggests that they execute different functions. For example, in this review, based on recent findings, we propose that vestibular acceleration signals are used for heading estimation, while velocity signals are used for computing displacement or distance traveled. The reliance of the brain on different components for different functions could be due to adaptation to the environment. Through long-term evolution, the brain may have developed a strategy to sense instantaneous self-motion direction as rapidly as possible by using the quick acceleration signal, for example, when the animal is pursuing prey or escaping from predators. In any case, future studies should be conducted to understand the functional implications of different vestibular temporal components, including velocity, acceleration, and jerk, as well as their interactions with signals in other sensory modalities, including vision, proprioception, and audition.
